# Genomic and Antimicrobial Resistance Analysis of an ST25 *Streptococcus suis* Strain Isolated from a Human in Zhejiang Province, China

**DOI:** 10.3390/pathogens14080742

**Published:** 2025-07-28

**Authors:** Shuirong Zhu, Xiaofang Wu, Wenwu Yao, Zhuoying Wu, Lingbo Wang, Zhangnv Yang, Beibei Wu, Yanjun Zhang

**Affiliations:** 1 Zhejiang Provincial Center for Disease Control and Prevention, Hangzhou 310051, China; 2 Huzhou Center for Disease Control and Prevention, Huzhou 313000, China

**Keywords:** *Streptococcus suis*, human isolate, ST25 sequence type, antimicrobial susceptibility testing, genomic analysis

## Abstract

A *Streptococcus suis* strain isolated from the blood of a patient in Zhejiang Province, China, was analysed using whole-genome sequencing and tested for antimicrobial resistance. The isolated strain was identified as *S. suis* serotype 2, and classified to ST25 on multilocus sequence typing (MLST). The minimum core genome group of the strain was identified as Group 4, and multilocus variable-number tandem-repeat analysis (MLVA) assigned it as type 2, 4.4, 0, 9, 3, 2, 0, 0. An antimicrobial resistance analysis showed that the strain was resistant to clindamycin, tetracycline, azithromycin, and erythromycin but sensitive to 11 other antibiotics. In a genomic evolution analysis, this isolate clustered on the same branch as North American pig isolate, Chinese pig isolates from Tianjin, and Hubei pig isolates.

## 1. Introduction

*Streptococcus suis* infection is a serious zoonotic disease affecting both humans and pigs. The clinical manifestations include meningitis, sepsis, and endocarditis, with high mortality rates and hearing loss as a sequela [1]. *S. suis* is currently classified into 29 serotypes based on the antigenicity of the capsular polysaccharide [2,3,4]. Among these, *S. suis* serotype 2 (SS2) is the most prevalent type and severely pathogenic in both humans and pigs. It frequently causes occupational contact infections in adults and is notably the predominant pathogenic serotype in Southeast Asian nations, including Thailand and Vietnam, as well as China [5]. SS2 was responsible for three outbreaks of human *S. suis* infection in China: Jiangsu Province (1998), Sichuan Province (2005), and Guangxi Province (2016) [6,7,8], which resulted in multiple infections and deaths, attracting public health attention. Multilocus sequence typing (MLST; https://pubmlst.org/ssuis/, accessed on 15 July 2025) has defined 3030 multilocus sequence types (STs) for *S. suis*, among which ST1 is prevalent worldwide (predominantly in Eurasia) [5,9,10], while ST7 has been reported only in China and Italy [11,12]. The ST7 strain caused the 1998 and 2005 outbreaks in China and carries the 89K pathogenicity island (89K PAI). All previously reported human infections in Zhejiang Province were attributed to SS2, including ST7 and ST1 [13]. In 2018, an ST25 SS2 strain was detected for the first time in Zhejiang Province. This sequence type is predominantly reported in North American pigs with high detection rates [11,14,15], suggesting specific, previously unnoticed changes in epidemic strains of *S. suis* in Zhejiang Province in recent years. Antibiotics plays an important role in treating *S. suis* infection in both species; monitoring the antimicrobial susceptibility of the ST25 strain may help optimize antibiotic therapy. We used the microdilution method to determine the minimum inhibitory concentration (MIC) for the strain. Moreover, whole-genome sequencing (second-generation combined with third-generation sequencing) was conducted to analyse virulence, antimicrobial resistance, and phylogeny.

## 2. Materials and Methods

### 2.1. Strains

The experimental strain ZJSS31 was isolated from a sporadic human *S. suis* infection case and submitted to the Huzhou Center for Disease Control and Prevention, Zhejiang Province (2018). Genomic sequences of the reference strains were obtained from GenBank, including: the highly virulent strain SS2 ST1 strain P1/7 (GCA_000091905.1), moderately virulent ST25 strain 85-1591 (GCA_000167375.1) and NSUI060 (GCA_001572685.1), and the low/avirulent ST28 strain NSUI002 (GCA_001272635.1).

### 2.2. Case Information

A 51-year-old male working in raw pork sales developed a fever of unknown origin on 2 August 2018. He denied headache, nausea, vomiting, abdominal pain, or diarrhoea. On 5 August, he presented to a private clinic with a body temperature of 39 °C and was referred to a Zhejiang Province hospital for suspected infectious fever. Physical examination revealed no skin bleeding spots, petechiae, or ecchymoses. His maximum temperature was 39.5 °C without nuchal stiffness, toxic shock syndrome, respiratory distress syndrome, or meningeal irritation signs. 6 August laboratory tests showed: white blood cell (WBC) count 15.8 × 10^9^/L, neutrophil (N) 81.6%, lymphocyte (L) 12.9%, and platelets (PLTs) 164 × 10^9^/L. *S. suis* was isolated from bacterial culture (8 August). He received anti-infective and supportive therapy; follow-up tests on 9 August showed: WBC count 9.4 × 10^9^/L, N 61.7%, L 28.4%, PLT 241 × 10^9^/L, and normal coagulation, liver, and kidney function. The patient recovered and was discharged on 16 August.

### 2.3. Reagents, Instruments, and Strain Identification

Columbia blood agar (Oxoid, Basingstoke, UK)*, S. suis* antiserum (Statens Serum Institut, Copenhagen, Denmark), and *Streptococcus* susceptibility plates (Shanghai Thermo Fisher Scientific, Shanghai, China) were used. Instruments used in this study included: VITEK 2 Compact advanced system (v05.01; bioMérieux, Marcy-l’Étoile, France), VITEK MS mass spectrometer (V2.3.3; bioMérieux, Marcy-l’Étoile, France), carbon dioxide (CO_2_) incubator (Thermo, Waltham, MA, USA), and Mastercycler gradient PCR and centrifuge (Eppendorf Company, Hamburg, Germany).

Single colonies were picked from blood agar and incubated (37 °C, 5% CO_2_ 18–24 h). Strain identification was performed by Gram staining, the VITEK 2 Compact and VITEK MS. Serotyping used *S. suis* type 1/2 antisera in a slide agglutination test (normal saline control). Genus, species, serotype-specific genes, virulence genes, and genotyping were determined via PCR as previously published [13,16].

### 2.4. Antimicrobial Susceptibility Testing (AST)

AST was performed using the microdilution method for azithromycin, cefepime, cefotaxime, ceftriaxone, chloramphenicol, clindamycin, daptomycin, ertapenem, erythromycin, levofloxacin, linezolid, meropenem, penicillin, tetracycline, and vancomycin (the microdultion of the antimicrobial agents is shown in Supplementary Figure S1). The American Clinical and Laboratory Standards Institute (CLSI, M100-S23) breakpoints for *S. viridan*in were applied [17]. *S. pneumoniae* ATCC 49619 was the quality control strain for AST. The protocol is listed briefly as follows: After culturing at 37 °C under 5% CO_2_ for 16–18 h, single colonies were picked and suspended in 5 mL of CAMHBT Broth to 0.5 McFarland standard. Then 100 μL suspension was added to 11 mL of Mueller–Hinton (MH) broth containing horse blood. Aliquots of the MH broth suspension (100 μL) were transferred to susceptibility plates according to the manufacturer’s protocol. Plates were sealed with adhesive film and incubated (37 °C, non-CO_2_, 24 h) before reading.

### 2.5. Genome Sequencing and Assembly

Both second- and third-generation sequencing were performed by Zhejiang Tianke High-tech Development Co., Ltd. (Hangzhou, China). (1) DNA extraction: Genomic DNA was extracted using Gentra Puregene Yeast/Bact. Kit (Qiagen, Redwood, CA, USA). DNA concentration was measured (NanoDrop™ 2000, Thermo Scientific, Waltham, MA, USA) and verified via agarose gel electrophoresis. An amount of >5 μg of DNA was used for library preparation. (2) Sequencing: Libraries were prepared with TruePrep™ DNA Library Prep Kit V2 (Vazyme, Nanjing, China) and the Ligation Sequencing Kit (SQ-LSK109, Oxford Nanopore, Oxford, UK). Sequencing was conducted on the Illumina NovaSeq platform (Illumina, San Diego, CA, USA) and the GridION X5 platform (Oxford Nanopore, UK). (3) Assembly: Canu v1.8 (https://canu.readthedocs.io/en/latest/index.html, accessed on 25 July 2025) processed raw reads from both platforms to generate high-quality data. Unicycler v0.4.5 (https://github.com/rrwick/Unicycler, accessed on 25 July 2025) assembled Illumina reads and Nanopore reads into a complete genome. Circos (v0.69) was used to plot the genome.

### 2.6. Bioinformatic Analysis

We downloaded genomic sequences of 34 ST25 strains from the National Center for Biotechnology Information (NCBI) database (Table 1). A whole-genome SNPs-based phylogenetic tree of ZJSS31 strain and these strains was constructed using SplitsTree4.

The ST of *S. suis* isolates was determined using multilocus sequence typing software (https://www.pubmlst.org, accessed on 15 July 2025). Antimicrobial-resistant genes and plasmid profiles were analysed using ResFinder (https://cge.food.dtu.dk/services/ResFinder/, accessed on 13 October 2024) and PlasmidFinder (https://cge.food.dtu.dk/services/PlasmidFinder/, accessed on 13 October 2024). The detection of virulence genes was performed using VFDB online database10 (http://www.mgc.ac.cn/VFs/, accessed on 13 October 2024). Annotation of mobile elements was carried out using online databases, such as ISfinder (https://www-is.biotoul.fr/credits.php, accessed on 15 July 2025). Plasmid sequence alignment was performed using Easyfig v2.2.5 (https://mjsull.github.io/Easyfig/, accessed on 13 October 2024).

## 3. Results

### 3.1. Strain Identification

Alpha haemolysis was evident surrounding the colonies. Gram staining revealed Gram-positive cocci arranged in pairs or short chains under oil immersion microscope. Matrix-assisted laser desorption/ionisation time-of-flight mass spectrometry (MALDI–TOF-MS) identification confirmed the strain to be *S. suis*, with agglutination observed for SS2 diagnostic antiserum but not SS1 antiserum. No agglutination occurred in the saline control. PCR [13,16] identified the genotype as MLST/ST25, MCG/Group 4, and MLVA/(2, 4.4, 0, 9, 3, 1, 2, 0, 0). This strain is hereafter designated ‘ZJSS31/ST25’.

### 3.2. Antimicrobial Susceptibility Testing (AST)

The AST of the strain showed resistance to clindamycin, tetracycline, azithromycin, and erythromycin, but sensitivity to 11 other antimicrobial agents: cefepime, cefotaxime, ceftriaxone, chloramphenicol, daptomycin, ertapenem, levofloxacin, linezolid, meropenem, penicillin, and vancomycin (Table 2).

### 3.3. Comparison of Genomic Sequences

**Figure 1:**
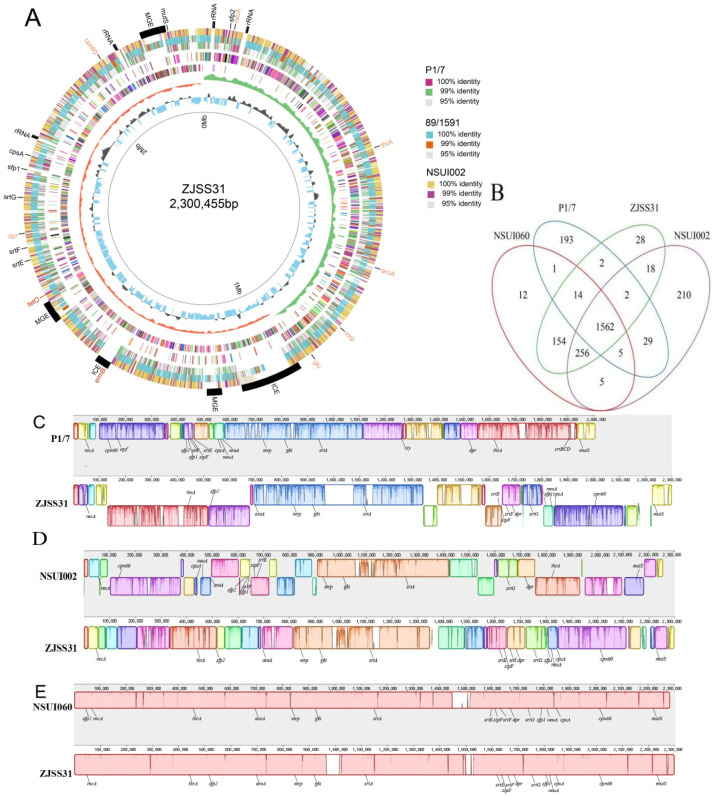
Whole genome analysis of ZJSS31 and genomic comparison with P1/7, NSUI002, and NSUI060. (**A**) Circular genome representation (inner to outer rings): 1. Genome scale; 2. GC content; 3. GC skew (green represents the positive and orange represents negative); 4. CDSs on negative strand, and different colours represent different clusters of orthologous genes (COG) annotation categories of the CDSs; 5. CDSs on the positive strand, and different colours represent the different COG annotation categories of the CDSs; 6. TBLASTN results with strain P1/7; 7. TBLASTN results with strain 89/1591; 8. TBLASTN results with strain NSUI002; 9. Annotation of key features: capsule- and pilus-related genes, rRNA, mobile genetic elements (MGEs) (marked in orange), drug-resistance genes (marked in red), and virulence genes (marked in blue). (**B**) Venn diagram of shared gene clusters among ZJSS31, P1/7, NSUI002, and NSUI060s. (**C**) Collinearity comparison between ZJSS31 genome and P1/7 genome (ProgressiveMauve). (**D**) Collinearity comparison between the ZJSS31 genome and the NSUI002 genome. (**E**) Collinearity comparison between the ZJSS31 genome and NSUI060 genome.

The complete genome of ZJSS31/ST25 (Genbank accession number: 2813098) was 2,300,455 bp (GC content: 41.02%), encoding four 5S/16S/23S rRNAs, 57 tRNAs, 42 ncRNAs, 2351 open reading frames (ORF), 1912 coding sequences (CDSs), and 17 pseudogenes. The genome is enriched with insertion sequences (IS), transposons (e.g., Tn917), prophages, and integrative conjugative elements (ICEs). Five large mobile genetic elements (MGEs) were identified: three unclassified MGEs and two integrative and conjugative elements (ICEs), one carrying the *erm*B macrolide-resistance gene. Insertion sequence (IS) elements included ICESsuYS66, IMESsuYS67, IS4, ISL3, IS982, IS110, IS200, IS605, IS630, and IS30. Pilus genes (*srt*A, *srt*E, *srt*F, *srt*G, *sfp*1, *sfp*2, *sip*F) and virulence genes (lysozyme-releasing protein gene *mrp*, capsular polysaccharide gene *cps*A) were detected (Figure 1A). Nine prophages and a plamid of 5581 bp were predicted.

Orthology analysis showed ZJSS31/ST25 shares: 1562 gene clusters with strains P1/7/ST1, NSUI002/ST28, and NSUI060/ST25; 1986 gene clusters with strain NSUI060/ST25; 1838 gene clusters with strain NSUI002/ST28; and 1580 gene clusters with P1/7/ST1 (Figure 1B).

Genome collinearity revealed ZJSS31/ST25′s genome size was similar to NSUI002/ST28 and NSUI060/ST25 but larger than P1/7/ST1 (Figure 1C–E). ZJSS31/ST25 showed the highest homology with NSUI060/ST25. Unlike P1/7/ST1 (which carries the *srtBCG*, mrp, the extracellular protein factor gene *epf*, and the hemolysin virulence gene *sly*), ZJSS31/ST25, NSUI002/ST28, and NSUI060/ST25 possess only *mrp* and *srt*G pilus island. Chromosomal rearrangements were observed in pilus islands and *cps*A loci across strains.

### 3.4. Antibiotic-Resistance Genes, ICEs, and Phylogeny

**Figure 2:**
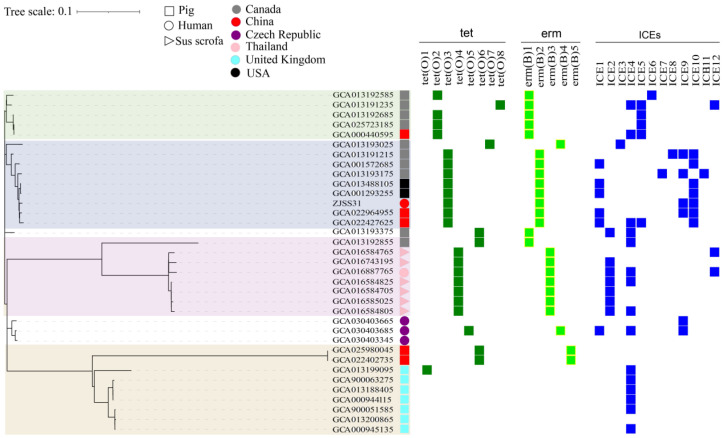
Phylogenetic, antimicrobial resistance, and ICE analyses of 35 ST25 strains of *S. suis.* Phylogenetic tree showing genetic relationship, with branches colour-coded by geographic clade, Canada (green), North America (blue), Thailand (red), and the UK (yellow). The right side of the figure describes the host, country of origin, and presence of ICEs and/or resistance genes. Strains isolated from pigs are represented by squares; strains isolated from wild pigs are represented by triangles, and those isolated from humans are represented by circles. Strains isolated from different countries are shown in different colours: grey for Canada, purple for Czech Republic, pink for Thailand, light blue for the UK, and black for the USA. Tetracycline-resistance genes are shown in green, and erythromycin-resistance genes in light green. ICEs are shown in dark blue.

ZJSS31/ST25 carries the tetracycline-resistance *tet*(O) gene and allelic macrolide-resistance gene *erm*B, like Chinese (Tianjin: GCA_022964955.1; Hubei: GCA_022427625.1) and North American pig strains (two strains from the United States and three strains from Canada). It also harbours two drug-resistance genes (*pat*A and *pat*B) and allelic insertion elements (ICEs9/10). The ICE distribution varied among strains as shown in Figure 2.

A neighbour-joining phylogenetic tree of 35 ST25 strains (based on 2155 SNPs) revealed four clades: United Kingdom, Thailand, North America, and Canada. ZJSS31/ST25 clustered within the North American clade but showed closest affinity to Chinese pig strains from Tianjin and Hubei, China.

## 4. Discussion

The diverse and sporadic human *S. suis* strains pose public health risks [18]. Without vaccines, antimicrobial treatment remains critical. Consequently, the prompt surveillance of antimicrobial resistance in *S. suis* is a critical global concern. In this study, we tested the MICs of 15 antibacterial agents for strain ZJSS31/ST25, and found that the strain is resistant to clindamycin, tetracycline, azithromycin, and erythromycin (the antibiotic type belongs to the lincosamide, tetracycline, and macrolide classes), and is sensitive to the other antibacterial agents tested. This contradicts findings of Athey et al. [15], which indicated that SS2/ST25 strains are mainly resistant to tetracycline and erythromycin. However, it is consistent with Nedbalcova et al. also reporting that *S. suis* is resistant to more than two types of antibiotics, including clindamycin and tetracycline [19,20,21]. High-level antimicrobial resistance (AMR) to tetracyclines, macrolides, and lincosamides has been reported in both human and swine isolates of *S. suis* [22,23,24]. As the ARGs (antibiotic resistance genes) *tet*(O) and *erm*B are the most common genes associated with AMR, monitoring antibiotic sensitivity will help to optimize antibiotic therapy in humans and swine.

Next-generation sequencing is a potent diagnostic technique for detecting bacterial infections [25]. Whole-genome sequencing has emerged as an essential and robust tool for investigating the genetic diversity of *S. suis*, identifying novel sequence types, and determining virulence factors [26]. At present, these technologies are the principal means of evaluating the genetic similarity of isolates sourced from various origins and have been extensively used to categorize and investigate the genetic relationship of *S. suis* across diverse regions and hosts. In this study, we used second- and third-generation sequencing to analyse strain ZJSS31/ST25, and found that it carries two drug-resistance genes, *tet*(O) and *erm*B, which confer resistance to tetracyclines and macrolides, respectively. *Tet*(O) is located on the original chromosome, whereas *erm*B is located on an ICE, suggesting the horizontal acquisition of the *erm*B. *Tet*(O) and *erm*B are the primary resistance genes identified in strains resistant to tetracycline and macrolides [26]. The sequencing results showed that antibiotic resistance genes in strain ZJSS31/ST25 were consistent with AST and literature reports. While *pat*A and *pat*B, which encode the ABC transporter associated with fluoroquinolone resistance, were also detected in strain ZJSS31/ST25 (60% sequence similarity between ZJSS31/ST25 sequence and the reference sequences)*,* no phenotypic resistance was observed in AST. Interestingly, the AST showed that ZJSS31/ST25 is resistant to clindamycin, but this lacks a genetic explanation.

The acquisition of antibiotic resistance facilitated by ICEs carrying antibiotic resistant genes in zoonotic pathogens poses a significant public health concern. Various *Streptococcus* species share similar ICE insertion sites, potentially allowing interspecies ICE integration [24]. Research has indicated that *S. suis* ICEs can undergo recombination with ICEs originating from other *streptococcal* species, such as *S. agalactiae* and *S. pyogenes* [27]. The ZJSS31/ST25 genome harbours five MGEs, including diverse IS elements and prophages. It is clear that strains under environmental selective pressures frequently acquire or passively adapt to incorporate foreign DNA gene segments. This phenomenon can lead to genetic heterogeneity within populations of the strain during its evolution. ZJSS31/ST25 and P1/7/ST1 carry different virulence genes. The former only carries one virulence gene, *mrp*, whereas three major virulence genes were detected in the latter, *mrp*, *epf*, and *sly*, which are the major virulence genes of *S. suis*. However, the highly virulent 89 K pathogenic island found in the strains responsible for the Chinese outbreaks was not detected in either strain. The significance of the pilus in *S. suis* virulence remains contentious [28,29]. Strain ZJSS31/ST25 also differs from strain P1/7/ST1 in terms of the pilus islands or clusters carried. Specifically, the former contains the srtG pilus island but lacks the srtBCG pilus cluster, whereas the latter has the reverse configuration. The presence of the srtBCG pilus cluster differs significantly between virulent and avirulent strains [30] insofar as it is present in all highly virulent strains but not in any avirulent strain. Therefore, the virulence of the Chinese human strain ZJSS31/ST25 is weaker than that of P1/7/ST1, which is also a moderately virulent strain, belonging to the same cluster as the NSUI060/ST25 pig strain [14]. The capsular polysaccharide is a major virulence factor and has been proposed as a suitable antigen for vaccinology and molecular typing [31]. Both ZJSS31/ST25 and the reference strains carry the *cps*A gene. There are gene rearrangements in the pilus islands and at the *cps*A locus in the reference strains. The causality and effects of these changes require further study.

A collinearity analysis indicated that the genome size of strain ZJSS31/ST25 is close to NSUI002/ST28 and NSUI060/ST25, but is much larger than that of virulent strain P1/7/ST1. This variation may stem from the presence of ICEs, transposons, plasmids, and prophages within strain ZJSS31/ST25. Orthology analysis revealed that the genome of strain ZJSS31/ST25 shares markedly more gene clusters with NSUI002/ST28 and NSUI060/ST25, indicating relatively close relationships among them. The genomes of avirulent strains tend to be larger than those of virulent strains, and avirulent strains typically carry more prophage sequences than virulent strains [32], confirming that Chinese human strain ZJSS31/ST25 is less virulent than strain P1/7/ST1 and may be a moderately virulent strain. This requires confirmation in future studies.

Phylogenetic analysis indicated 35 ST25 strains clustered into four major clades: UK, Thailand, North America, and Canada (the North American clade was subdivided into two subclades, North America and Canada), based on the allelic resistance genes, ICE types, and ICE abundances. ZJSS31/ST25 clusters with the North American strain, indicating a close relationship. Pig-derived strains of Tianjin and Hubei (GCA_022964955.1 and GCA_022427625.1) in China showed closer affinities with strain ZJSS31/ST25. This implies that the Chinese human-derived strain ZJSS31/ST25 shares homology with native pig-derived strains in China, in a same cluster with the lineage in North America.

SS2/ST25 strains are the most common serotype in North America (accounting for 44% of all reported strains) [27,33], yet human infections and deaths are rare in this region, which demonstrates differences in pathogenicity among different *S. suis* populations. A significant portion of the variance in disease occurrence and severity can be attributed partly to variations among strains. Cases involving ST25 strains were documented in Hong Kong (2007) and Shenzhen (2022), China [34,35]. The human isolate examined in this present study was first identified in Zhejiang Province in 2001. Therefore, understanding the antimicrobial resistance patterns, population structures, and genetic diversity of human-infecting strains will assist clinicians in rational drug use and aid epidemiology personnel in dealing with potentially virulent strains with significant genetic variability. In summary, pathogen studies enhance our understanding of their risks while providing a molecular basis for vaccine development and strain selection.

## 5. Limitations of the Study

This study focused on a single isolate, limiting broader epidemiological interpretations. Field validation requires more additional strains to confirm circulation patterns. The virulence of ZJSS31/ST25 was not assessed in animal models and requires further investigation.

## Figures and Tables

**Table 1:** Information on the 34 ST25 strains of *S. suis* used in this study.

Accession No.	Strain Name	Submission Time	Region	Host	Sequence Type	Serotype
GCA_022964955.1	TJS75	2015	Tianjin, China	Pig	ST25	2
GCA_025980045.1	WUSS030	2017	Jiangsu, China	Pig	ST25	8
GCA_022427625.1	LSSP102	2017	Hubei, China	Wild pig	ST25	2
GCA_022402735.1	WUSS030	2022	Nanjing, China	Pig	ST25	8
GCA_000440595.1	89-1591	2012	Beijing, China	Pig	ST25	2
GCA_001572685.1	NSUI060	2008	Canada	Pig	ST25	2
GCA_013192685.1	89-1591	1989	Canada	Pig	ST25	2
GCA_013192855.1	1636820	2014	Canada	Pig	ST25	2
GCA_013193025.1	1602951	2014	Canada	Pig	ST25	2
GCA_013191235.1	89-6891-2	1989	Canada	Pig	ST25	2
GCA_013192585.1	89-5046	1989	Canada	Pig	ST25	2
GCA_013191215.1	90-2741-7	1990	Canada	Pig	ST25	2
GCA_013193175.1	1667796	2014	Canada	Pig	ST25	2
GCA_013193375.1	1666725	2014	Canada	Pig	ST25	2
GCA_025723185.1	89-1591	1989	Canada	Pig	ST25	2
GCA_001293255.1	ISU2514	2014	USA	Wild pig	ST25	2
GCA_013488105.1	ISU2514	2014	USA	Wild pig	ST25	2
GCA_030403345.1	150002	2004	Czech	Human	ST25	2
GCA_030403665.1	148897	2003	Czech	Human	ST25	2
GCA_030403685.1	143741	2001	Czech	Human	ST25	2
GCA_900063275.1	SS1053	2012	UK	Pig	ST25	2
GCA_000944115.1	S15T	2010	UK	Pig	ST25	2
GCA_900051585.1	SS1064	2012	UK	Pig	ST25	2
GCA_000945135.1	S16C	2010	UK	Pig	ST25	2
GCA_013199095.1	TMW_SS009	2013	UK	Pig	ST25	2
GCA_013188405.1	SS1053	2010	UK	Pig	ST25	2
GCA_013200865.1	SS1064	2010	UK	Pig	ST25	2
GCA_016743195.1	DP_SS29	2012	Thailand	Wild pig	ST25	2
GCA_016584705.1	MNCM02	2000	Thailand	Wild pig	ST25	2
GCA_016585025.1	MNCM34	2002	Thailand	Wild pig	ST25	2
GCA_016584825.1	MNCM13	2001	Thailand	Wild pig	ST25	2
GCA_016584765.1	MNCM03	2000	Thailand	Wild pig	ST25	2
GCA_016887765.1	ID30190	2009	Thailand	Human	ST25	2
GCA_016584805.1	MNCM11	2001	Thailand	Wild pig	ST25	2

**Table 2:** AST of the ZJSS31 isolate using a panel of 15 antimicrobial agents.

Antibiotic Type		Antimicrobial Agent	MIC (mg/L)/(R/I/S)
β-lactams	Penicillins	Penicillin	≤0.03 (S)
	Cephalosporins	Cefotaxime	≤0.12 (S)
		Ceftriaxone	≤0.12 (S)
		Cefepime	≤0.5 (S)
Carbapenems		Meropenem	≤0.25 (S)
		Ertapenem	≤0.5(S)
Tetracycline		Tetracycline	>8 (R)
Chloramphenicol		Chloramphenicol	=4 (S)
Macrolides		Azithromycin	>2 (R)
		Erythromycin	>2 (R)
Lincosamides		Clindamycin	>1 (R)
Cyclic lipopeptide		Daptomycin	=0.12 (S)
Quinolones		Levofloxacin	≤0.5 (S)
Oxazolidone		Linezolid	=1 (S)
Glycopeptides		Vancomycin	≤0.5 (S)

R, Resistant; I, Intermediate; S, Susceptiable.

## Data Availability

The data presented in this study are openly available in National Microbiology data center at https://www.ncbi.nlm.nih.gov/genbank, reference number 2813098 (accessed on 29 March 2024).

## Supplementary Materials

The following supporting information can be downloaded at: https://www.mdpi.com/article/10.3390/pathogens14080742/s1, Figure S1: The microdilution of antimicrobial agents in the *Streptococcus* susceptibility plate.

## References

[1] Tharavichitkul P., Wongsawan K., Takenami N., Pruksakorn S., Fongcom A., Gottschalk M., Khanthawa B., Supajatura V., Takai S. (2014). Correlation between PFGE Groups and mrp/epf/sly Genotypes of Human *Streptococcus suis* Serotype 2 in Northern Thailand. J. Pathog..

[2] Yongkiettrakul S., Maneerat K., Arechanajan B., Malila Y., Srimanote P., Gottschalk M., Visessanguan W. (2019). Antimicrobial susceptibility of *Streptococcus suis* isolated from diseased pigs, asymptomatic pigs, and human patients in Thailand. BMC Vet. Res..

[3] Zheng H., Ji S., Liu Z., Lan R., Huang Y., Bai X., Gottschalk M., Xu J. (2015). Eight Novel Capsular Polysaccharide Synthesis Gene Loci Identified in Nontypeable *Streptococcus suis* Isolates. Appl. Environ. Microbiol..

[4] Zheng H., Qiu X., Roy D., Segura M., Du P., Xu J., Gottschalk M. (2017). Genotyping and investigating capsular polysaccharide synthesis gene loci of non-serotypeable *Streptococcus suis* isolated from diseased pigs in Canada. Vet. Res..

[5] Feng Y., Zhang H., Wu Z., Wang S., Cao M., Hu D., Wang C. (2014). *Streptococcus suis* infection: An emerging/reemerging challenge of bacterial infectious diseases?. Virulence.

[6] Ye C., Zhu X., Jing H., Du H., Segura M., Zheng H., Kan B., Wang L., Bai X., Zhou Y. (2006). *Streptococcus suis* sequence type 7 outbreak, Sichuan, China. Emerg. Infect. Dis..

[7] Tang J., Wang C., Feng Y., Yang W., Song H., Chen Z., Yu H., Pan X., Zhou X., Wang H. (2006). Streptococcal toxic shock syndrome caused by *Streptococcus suis* serotype 2. PLoS Med..

[8] Huang W., Wang M., Hao H., Yang R., Xie J., Su J., Lin M., Cui Y., Jiang Y. (2019). Genomic epidemiological investigation of a *Streptococcus suis* outbreak in Guangxi, China, 2016. Infect. Genet. Evol..

[9] van Leengoed L.A., Vecht U., Verheyen E.R. (1987). *Streptococcus suis* type 2 infections in pigs in the Netherlands (Part two). Vet. Q..

[10] Schultsz C., Jansen E., Keijzers W., Rothkamp A., Duim B., Wagenaar J.A., van der Ende A. (2012). Differences in the population structure of invasive *Streptococcus suis* strains isolated from pigs and from humans in The Netherlands. PLoS ONE.

[11] Wang M., Du P., Wang J., Lan R., Huang J., Luo M., Jiang Y., Zeng J., Quan Y., Shi Z. (2019). Genomic Epidemiology of *Streptococcus suis* Sequence Type 7 Sporadic Infections in the Guangxi Zhuang Autonomous Region of China. Pathogens.

[12] Cucco L., Paniccià M., Massacci F.R., Morelli A., Ancora M., Mangone I., Di Pasquale A., Luppi A., Vio D., Cammà C. (2022). New Sequence Types and Antimicrobial Drug-Resistant Strains of *Streptococcus suis* in Diseased Pigs, Italy, 2017–2019. Emerg. Infect. Dis..

[13] Zhu S.R., Chen J.C., Wu Z.Y., Zhang Y.L., Fang L., Zhang Y.J. (2022). Molecular characteristics of *Streptococcus suis* type 2 from humans in Zhejiang Province. Chin. J. Infect. Dis..

[14] April A.A., Gottschalk M., Rossow S., Rendahl A., Gebhart C., Marthaler D.G. (2019). Serotype and Genotype (Multilocus Sequence Type) of *Streptococcus suis* Isolates from the United States Serve as Predictors of Pathotype. J. Clin. Microbiol..

[15] Athey T.B., Teatero S., Takamatsu D., Wasserscheid J., Dewar K., Gottschalk M., Fittipaldi N. (2016). Population Structure and Antimicrobial Resistance Profiles of *Streptococcus suis* Serotype 2 Sequence Type 25 Strains. PLoS ONE.

[16] Zhu S.R., Zhang Z., Yao P.P., Yang Y., Fang L., Zhang Y.J. (2021). Identification and characterization of the virulence of *Streptococcus suis* from human patients in Zhejiang Province, China. Chin. J. Zoonoses..

[17] (2024). Performance Standards for Antimicrobial Susceptibility Testing, 34th Informational Supplement.

[18] Hu Y., Fu S., Zou G., Kerdsin A., Chen X., Dong X., Teng L., Li J. (2021). Genome analysis provides insight into hyper-virulence of *Streptococcus suis* LSM178, a human strain with a novel sequence type 1005. Sci. Rep..

[19] Nedbalcova K., Kucharovicova I., Zouharova M., Matiaskova K., Kralova N., Brychta M., Simek B., Pecha T., Plodkova H., Matiasovic J. (2022). Resistance of *Streptococcus suis* Isolates from the Czech Republic during 2018–2022. Antibiotics.

[20] Bojarska A., Molska E., Janas K., Skoczyńska A., Stefaniuk E., Hryniewicz W., Sadowy E. (2016). *Streptococcus suis* in invasive human infections in Poland: Clonality and determinants of virulence and antimicrobial resistance. Eur. J. Clin. Microbiol. Infect. Dis..

[21] Bamphensin N., Chopjitt P., Hatrongjit R., Boueroy P., Fittipaldi N., Gottschalk M., Kerdsin A. (2021). Non-Penicillin-Susceptible *Streptococcus suis* Isolated from Humans. Pathogens.

[22] Gurung M., Tamang M.D., Moon D.C., Kim S.R., Jeong J.H., Jang G.C., Jung S.C., Park Y.H., Lim S.K. (2015). Molecular basis of resistance to selected antimicrobial agents in the emerging zoonotic pathogen *Streptococcus suis*. J. Clin. Microbiol..

[23] Ichikawa T., Oshima M., Yamagishi J., Muramatsu C., Asai T. (2020). Changes in antimicrobial resistance phenotypes and genotypes in *Streptococcus suis* strains isolated from pigs in the Tokai area of Japan. J. Vet. Med. Sci..

[24] Aradanas M., Poljak Z., Fittipaldi N., Ricker N., Farzan A. (2021). Serotypes, virulence-associated factors, and antimicrobial resistance of *Streptococcus suis* isolates recovered from sick and healthy pigs determined by whole-genome sequencing. Front. Vet. Sci..

[25] Hayashi T., Tsukagoshi H., Sekizuka T., Ishikawa D., Imai M., Fujita M., Kuroda M., Saruki N. (2019). Next-generation DNA sequencing analysis of two *Streptococcus suis* ST28 isolates associated with human infective endocarditis and meningitis in Gunma, Japan: A case report. Infect. Dis..

[26] Raberahona M., Rasoanandrasana S., Rahajamanana V.L., Ranaivo-Rabetokotany F., Andriananja V., Rakotomalala F.A., Randria M.J.D., Rakotovao L., Marois-Créhan C., Tocqueville V. (2018). Novel *Streptococcus suis* Sequence Type 834 among Humans, Madagascar. Emerg. Infect. Dis..

[27] Palmieri C., Magi G., Mingoia M., Bagnarelli P., Ripa S., Varaldo P.E., Facinelli B. (2012). Characterization of a *Streptococcus suis* tet(O/W/32/O)-carrying element transferable to major streptococcal pathogens. Antimicrob. Agents Chemother..

[28] Marini E., Palmieri C., Magi G., Facinelli B. (2015). Recombination between *Streptococcus suis* ICESsu32457 and *Streptococcus agalactiae* ICESa2603 yields a hybrid ICE transferable to *Streptococcus pyogenes*. Vet. Microbiol..

[29] Fittipaldi N., Takamatsu D., de la Cruz Domínguez-Punaro M. (2010). Mutations in the gene encoding the ancillary pilin subunit of the *Streptococcus suis* srtF cluster result in pili formed by the major subunit only. PLoS ONE.

[30] Okura M., Osaki M., Fittipaldi N., Gottschalk M., Sekizaki T., Takamatsu D. (2011). The minor pilin subunit Sgp2 is necessary for assembly of the pilus encoded by the srtG cluster of *Streptococcus suis*. J. Bacteriol..

[31] Guo G., Du D., Yu Y., Zhang Y., Qian Y., Zhang W. (2021). Pan-genome analysis of *Streptococcus suis* serotype 2 revealed genomic diversity among strains of different virulence. Transbound. Emerg. Dis..

[32] Uruén C., Fernandez A., Arnal J.L., Del Pozo M., Amoribieta M.C., de Blas I., Jurado P., Calvo J.H., Gottschalk M., González-Vázquez L.D. (2024). Genomic and phenotypic analysis of invasive *Streptococcus suis* isolated in Spain reveals genetic diversification and associated virulence traits. Vet. Res..

[33] Fittipaldi N., Xu J., Lacouture S., Tharavichitkul P., Osaki M., Sekizaki T., Takamatsu D., Gottschalk M. (2011). Lineage and virulence of *Streptococcus suis* serotype 2 isolates from North America. Emerg. Infect. Dis..

[34] Chu Y.W., Cheung T.K., Chu M.Y., Tsang V.Y., Fung J.T., Kam K.M., Lo J.Y. (2009). Resistance to tetracycline, erythromycin and clindamycin in *Streptococcus suis* serotype 2 in Hong Kong. Int. J. Antimicrob. Agents..

[35] Ji L., Chen Z., Li F., Hu Q., Xu L., Duan X., Wu H., Xu S., Chen Q., Wu S. (2023). Epidemiological and genomic analyses of human isolates of *Streptococcus suis* between 2005 and 2021 in Shenzhen, China. Front. Microbiol..

