# Supplementary figures and images for: Genomic and Antimicrobial Resistance Analysis of an ST25 Streptococcus suis Strain Isolated from a Human in Zhejiang Province, China
Source: Pathogens. 2025 Jul 28;14(8):742. doi: 10.3390/pathogens14080742 (PMC12389401; doi:10.3390/pathogens14080742)

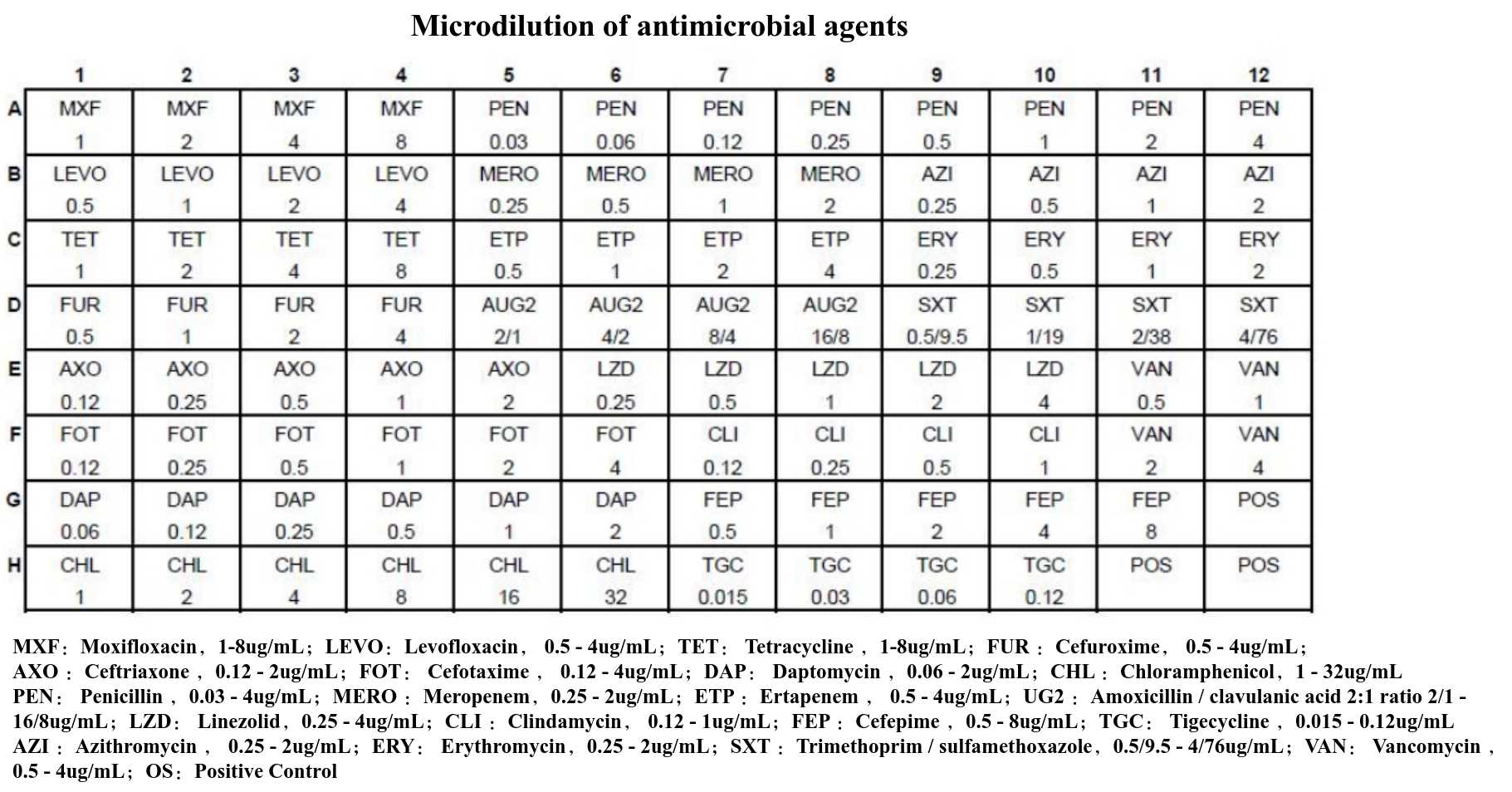

Supplement: Supplementary file 1 [file pathogens-14-00742-s001.zip › pathogens-3697198-supplementary.jpg]
